# Potential vectors of *Leishmania* spp. in an Atlantic Forest conservation unit in northeastern Brazil under anthropic pressure

**DOI:** 10.1186/s13071-020-04523-2

**Published:** 2021-01-11

**Authors:** Marcos Paulo Gomes Pinheiro, Cássio Lázaro Silva-Inacio, Marcel Miranda de Medeiros Silva, Paulo Sérgio Fagundes de Araújo, Maria de Fátima Freire de Melo Ximenes

**Affiliations:** Laboratório de Pesquisas em Entomologia, Departamento de Microbiologia e Parasitologia, Centro de Biociências, Universidade Federal do Rio Grande do Norte, Avenida Senador Salgado Filho, 3000 Natal, Rio Grande do Norte Brazil

**Keywords:** Phlebotomines, Seasonality, Atlantic Forest, *Lutzomyia longipalpis*, *Evandromyia walkeri*, *Psychodopygus wellcomei*

## Abstract

**Background:**

Phlebotomines are a group of insects which include vectors of the *Leishmania* parasites that cause visceral leishmaniasis (VL) and cutaneous leishmaniasis (CL), diseases primarily affecting populations of low socioeconomic status. VL in Brazil is caused by *Leishmania infantum*, with transmission mainly attributed to *Lutzomyia longipalpis*, a species complex of sand fly, and is concentrated mainly in the northeastern part of the country. CL is distributed worldwide and occurs in five regions of Brazil, at a higher incidence in the north and northeast regions, with etiological agents, vectors, reservoirs and epidemiological patterns that differ from VL. The aim of this study was to determine the composition, distribution and ecological relationships of phlebotomine species in an Atlantic Forest conservation unit and nearby residential area in northeastern Brazil.

**Methods:**

Centers for Disease Control and Shannon traps were used for collections, the former at six points inside the forest and in the peridomestic environment of surrounding residences, three times per month for 36 months, and the latter in a forest area, once a month for 3 months. The phlebotomines identified were compared with climate data using simple linear correlation, Pearson’s correlation coefficient and cross-correlation. The estimate of ecological parameters was calculated according to the Shannon-Wiener diversity index, standardized index of species abundance and the dominance index.

**Results:**

A total of 75,499 phlebotomines belonging to 11 species were captured in the CDC traps, the most abundant being *Evandromyia walkeri*, *Psychodopygus wellcomei* and *Lu. longipalpis*. *Evandromyia walkeri* abundance was most influenced by temperature at collection time and during the months preceding collection and rainfall during the months preceding collection. *Psychodopygus wellcomei* abundance was most affected by rainfall and relative humidity during the collection month and the month immediately preceding collection time. *Lutzomyia longipalpis* abundance showed a correlation with temperature and the rainfall during the months preceding collection time. The Shannon trap contained a total of 3914 phlebotomines from these different species. *Psychodopygus wellcomei*, accounting for 91.93% of the total, was anthropophilic and active mainly at night.

**Conclusions:**

Most of the species collected in the traps were seasonal and exhibited changes in their composition and population dynamics associated with local adaptions. The presence of vectors *Ps. wellcomei* and * Lu. longipalpis* underscore the epidemiological importance of these phlebotomines in the conservation unit and surrounding anthropized areas. Neighboring residential areas should be permanently monitored to prevent VL or CL transmission and outbreaks.
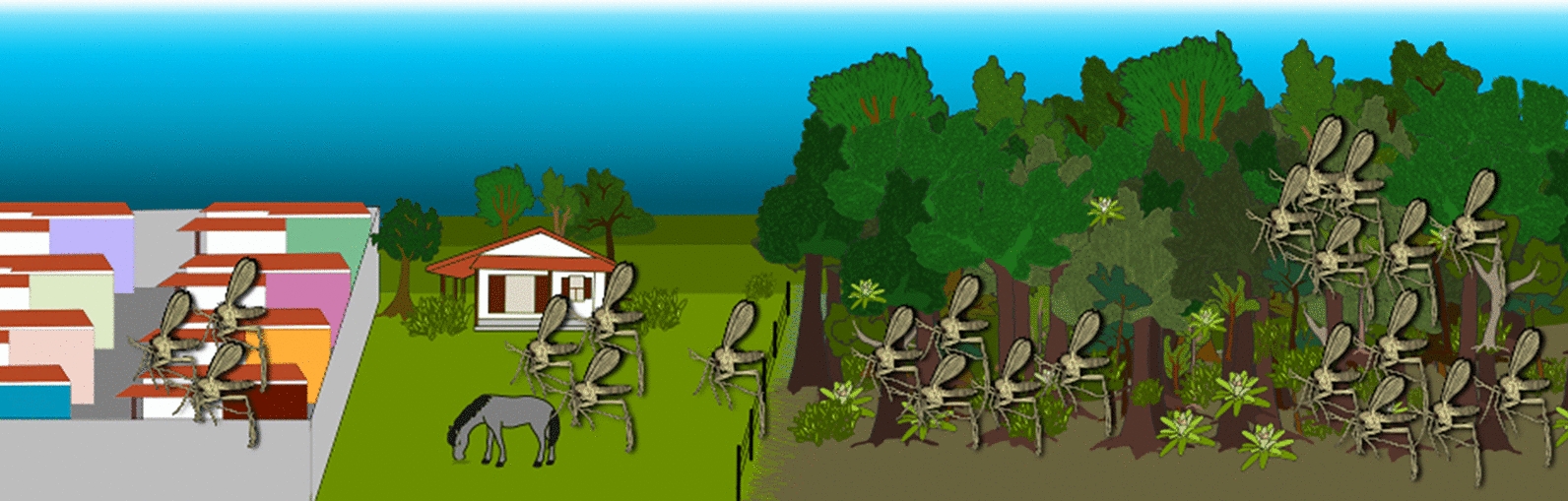

## Background

The leishmaniases are a group of diseases caused by several species of etiologic agents of the genus *Leishmania*. The two main forms of leishmaniasis are visceral leishmaniasis (VL) and cutaneous leishmaniasis (CL) [1, 2]. Phlebotomines (Diptera: Psychodidae) are small insects, and the hematophagous females of some phlebotomine species are responsible for transmitting protozoan parasites belonging to the genus *Leishmania*, known vectors of the leishmaniases, resulting in diseases that exhibit a series of clinical manifestations with a potential risk of death. The leishmaniases are considered to be neglected tropical diseases and are related to premature deaths and incapacitating lesions in several countries of the world [3–5].

In Brazil, VL is caused by *Leishmania infantum*, whose main vector is the phlebotomine *Lutzomyia longipalpis* [1], while CL has different vectors and etiologic agents, the principal vectors being *Nyssomyia whitmani*,* Nyssomyia intermedia*, *Nyssomyia neivai*, *Migonemyia migonei* and *Psychodopygus wellcomei* [2, 5–8]. *Leishmania braziliensis* is the most widely distributed etiologic agent in the country and is the primary culprit in cases of CL in northeastern Brazil [5].

Although leishmaniases occur in all regions of Brazil, VL cases are concentrated in the northeastern parts, with approximately 45% of all reported cases in 2017 occurring in this region. The northeast and northern regions continue to report a large number of CL cases [9, 10]. Studies show that the presence of settlements in forested areas and environmental degradation are important drivers of the rising number of leishmaniasis cases since they can contribute to the adaptation and expansion of sand fly vectors in areas of human intervention, whether in urban or periurban areas [11–14].

Our research group has observed changes in the composition and population dynamics of phlebotomines in Atlantic Forest fragments since the 1990s, where *Lutzomyia longipalpis* is the main phlebotomine species in the area. [15, 16]. The forest harbors wild mammals considered to be parasite reservoirs and is situated in the primary area of VL occurrence in the state and metropolitan region of Natal. In recent years, urbanization has increased in surrounding areas, with increasing numbers of families and their pets, mainly dogs.

The aim of this study was to conduct a long-term study in an endemic area of VL and CL and analyze the abundance, composition and ecological relationships among phlebotomine species in an Atlantic Forest conservation unit under anthropic pressure.

## Methods

### Study site

**Fig. 1:**
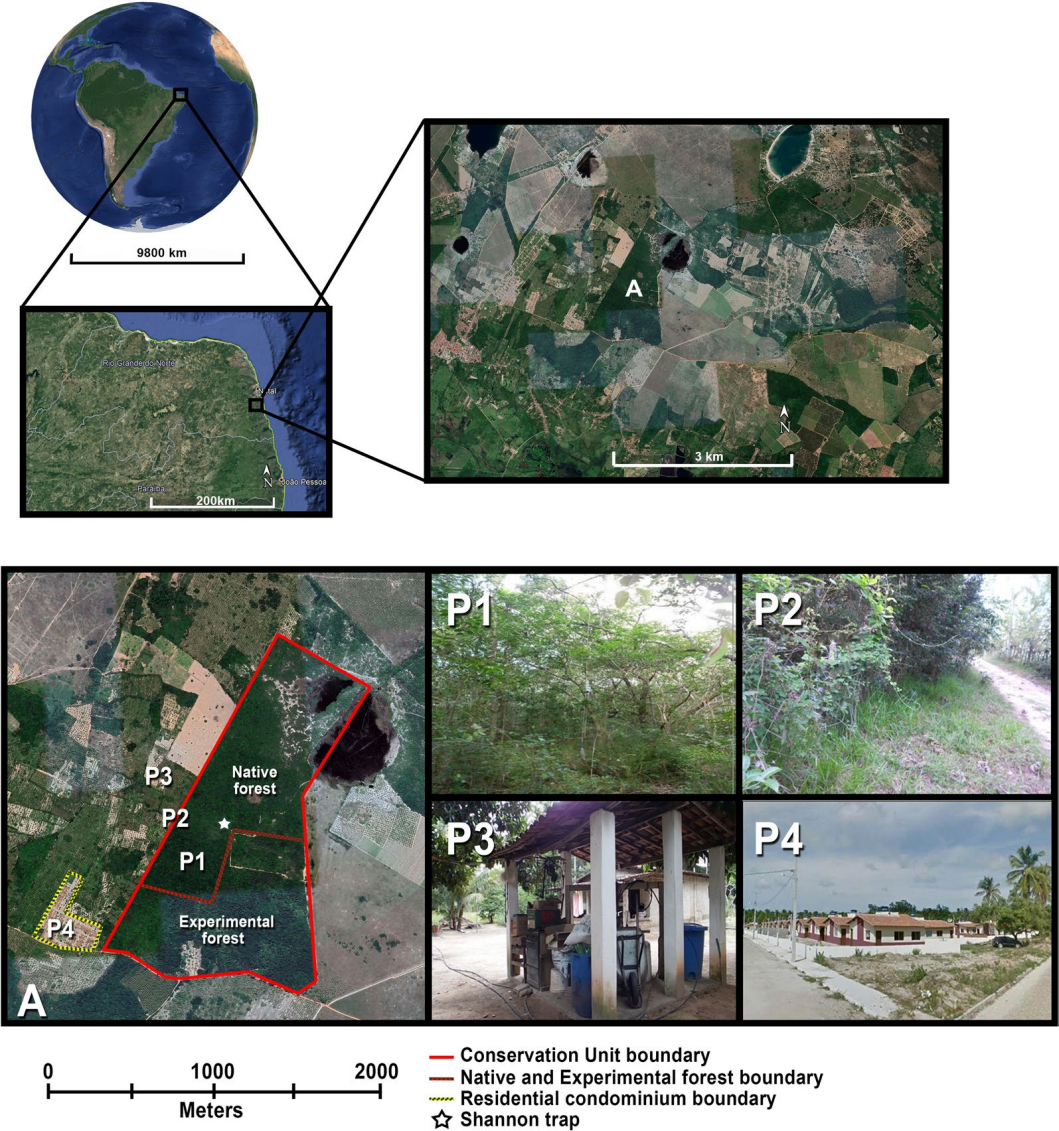
Atlantic Forest conservation unit (*A*) in the municipality of Nísia Floresta, Rio Grande do Norte State, Brazil, where the study took place. U.S. Center of Disease Control traps were placed at three collection points in the native forest area (*P1*) at a height of 12, 6 and 1 m, respectively, at the forest boundary (*P2*), in the rural peridomestic environment (*P3*) and in the peridomestic environment of a residential condominium (*P4*). The star indicates the Shannon trap collection point in the native forest

The study was conducted in a fragment of forest located in the Nísia Floresta National Forest (6°05′12.4′′S; 35°11′04.0′′W), an Atlantic Forest conservation unit, and in a nearby residential area in the municipality of Nísia Floresta, Rio Grande do Norte State, northeastern Brazil (Fig. 1). The municipality has reported cases of CL and VL, has a population of 27,260 inhabitants and is located in the metropolitan region of Natal, the state capital [17] (Fig. 1).

The Nísia Floresta National Forest lies in a region of tourism and urban expansion. It covers 168.84 hectares divided into an area of experimental forests where exotic species occur and a preserved area with semi-deciduous forest and coastal tableland.

The fauna in the area exhibits a wide diversity of arthropods, amphibians, reptiles, birds and mammals [18]. Among the mastofauna found are *Cerdocyon thous* (crab-eating fox), a wild potential reservoir of *Leishmania infantum*, *Didelphis albiventris* (white-eared opossum), a wild potential reservoir of *L. infantum* and parasite host of *L. braziliensis*, in addition to rodent parasite hosts or potential reservoirs of *L. infantum* and *L. braziliensis* [19, 20].

### Phlebotomine sampling

#### Collections with U.S. Center of Disease Control traps

Collections with U.S. Center of Disease Control (CDC) traps occurred in the area inside and surrounding the conservation unit (CU) (Fig. 1), at the following points: (i) three vertical strata at the same site in the conserved forest, denominated point 1 (P1A, P1B, P1C) (6°4′57.45″S/35°11′5.80″W), where the seasonal forest is most conserved; (ii) at the boundary of the seasonal forest, denominated point 2 (P2) (6°5′2.93″S/35°11′17.06″W); (iii) in the rural peridomestic environment, denominated point 3 (P3) (6°4′56.26″S/35°11′13.62″W), including the peridomestic environment with domestic animals (e.g. chickens, dogs and horses); (iv) in a peridomestic environment consisting of a condominium with 263 residents, denominated point 4 (P4) (6°5′11.76″S/35°11′27.05″W), located adjacent to the CU (Fig. 1).

Collections took place three times each month for 36 months, from September 2013 to August 2016, using CDC light traps that operated for 14 consecutive hours, from 5 p.m. to 7 a.m. the following morning, totaling 7776 h of sampling effort. The six traps used were arranged at different heights, as follows: three at P1 (P1A: 12 m; P1B: 6 m; P1C: 1 m), one at P2 (1 m), one at P3 (1 m) and one at P4 (1 m) (Fig. 1).

#### Collections with Shannon trap

Collections with the Shannon trap [21] took place in the semi-deciduous seasonal forest (Fig. 1) using two 8W white lights to attract the phlebotomines, powered by 6V, 12A batteries. Three collections lasting 24 hours were made, starting at 4 pm and concluding at the same time the next day, in June, August and October 2016, totaling 72 h of sampling. In the study area, the sun rises around 5:15 a.m. and sets about 12 h later. We considered twilight as soft diffused light from the sky when the sun is below the horizon, either from daybreak to sunrise, or from sunset to nightfall.

The captures were made by six collectors (3 pairs), who alternated every 2 h and wore suitable protective clothing. The phlebotomines that landed in the trap or on the collectors were captured with a manual aspirator, stored in plastic vials that were replaced with new vials each hour, taken to the laboratory, mounted and identified.

All phlebotomines captured with CDC and Shannon traps were processed and mounted onto microscopic slides [22]. They were identified using the phylogenetic classification proposed by Galati [23] and stored in the Professor Adalberto Antônio Varela-Freire Entomological Collection of the Federal University of Rio Grande do Norte (preservation code CEAAVF/UFRN/DIP0001).

#### Data analysis

BioEstat 5.3 (Mamirauá Institute, Tefé, Brazil), a software for (bio)statistical analysis, was used in Pearson’s correlation coefficient and simple linear regression analysis, with the insects representing the dependent variable and rainfall, relative humidity and temperature the independent variables. Meteorological data were obtained from the National Institute of Meteorology (INMET) weather station [24], located 30 km from the CU, which is part of the same mesoregion and displays similar climate characteristics. For all regressions performed, the residuals confirmed the assumption regarding the errors of the linear regression model.

The total number of specimens and the total species at each collection site for both sexes were assessed with analysis of variance using the Kruskal-Wallis and Mann-Whitney tests to compare differences between two means.

Species abundance was compared to mean rainfall, temperature and relative humidity, first assessing the autocorrelation patterns of both time series using multiple regression analysis and then applying the cross-correlation test using the Paleontological Statistics Software Package (PAST 2.17c). Cross-correlation is useful in aligning two time series, one of which is delayed with respect to the other, as its peak occurs at the lag at which the two time series are best correlated [25, 26]. Thus, we used the test to deepen the analysis of the relationships between the meteorological variables and phlebotomine abundance.

The standardized index of species abundance (SISA) [27] was calculated to compare abundance between the species found, with values varying between zero and one, using Microsoft Office Excel 2013 (Microsoft Corp., Redmond, WA, USA). Collection point diversity was analyzed based on species richness and equitability, using the Shannon-Wiener diversity index [28].

Relative species frequency was used to establish a dominance rank (D), according to the categories established by Silveira Neto et al [29], with eudominant > 10%; dominant > 5–10%; subdominant > 2–5%; occasional = 1–2%; and rare < 1%. D% = (*i*/*t*) × 100, where* i* is the number of individuals of a species and* t* is the total number collected.

## Results

### Phlebotomine diversity and abundance in forest and anthropized areas

A total of 75,499 phlebotomines belonging to seven genera and 11 species were captured in the CDC light traps. In general, males were more abundant than females; however, there was no significant difference in the male:female ratio (Mann-Whitney U-test:* U* = 60,* Z* = 0.0328,* P* = 0.48).

**Fig. 2:**
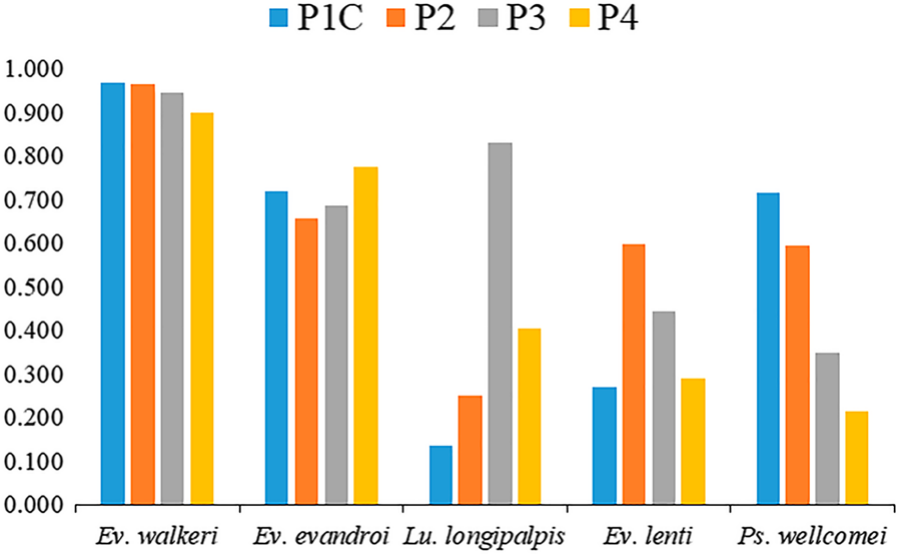
Standard index of species abundance (SISA) of the most abundant species collected, calculated for each ecotype analyzed.* P1C* conserved forest,* P2* forest boundary,* P3* rural environment,* P4* condominium complex

The most abundant species was *Evandromyia walkeri*, conributing to 79.7% of the phlebotomines collected (SISA = 1.00), primarily in the trap near the forest floor, where 21,039 *Ev. walkeri *individuals were captured (SISA = 0.969) (Fig. 2), followed in decreasing order of abundance by *Psychodopygus wellcomei* (12.7%; SISA = 0.89), *Evandromyia evandroi* (2.9%; SISA = 0.89) and *Lutzomyia longipalpis* (3.1%; SISA = 0.87) (Table 1).

**Table 1:** Species composition and abundance of phlebotomines captured in each ecotope (2013–2016)

Species composition and abundance	Ecotopes^a^						Total	Male:female ratio	Percentage	SISA
	Forest			Anthropized areas						
	P1A	P1B	P1C	P 2	P 3	P 4				
Species										
* Evandromyia walkeri*	9717	12,740	21,039	3260	11,725	1705	60,186	3.3:1	79.718	1.00
* Psychodopygus wellcomei*	1055	2411	4868	812	440	37	9623	0.4:1	12.746	0.89
* Evandromyia evandroi*	101	190	360	252	783	541	2227	1.9:1	2.950	0.89
* Lutzomyia longipalpis*	5	19	61	57	2102	106	2350	4.6:1	3.113	0.87
* Evandromyia lenti*	9	18	41	51	327	123	569	1.8:1	0.754	0.86
* Psathyromyia brasiliensis*	36	118	139	9	2	3	307	1.9:1	0.407	0.86
* Psathyromyia abonnenci*	19	23	22	4	32	2	102	0.8:1	0.134	0.86
* Sciopemyia sordellii*	8	10	18	24	13	3	76	0.2:1	0.101	0.86
* Micropygomyia schreiberi*	2	8	17	15	10	4	56	2.3:1	0.074	0.85
* Nyssomyia whitmani*	0	0	2	0	0	0	2	1.0:1	0.003	0.02
* Evandromyia sallesi*	0	0	0	1	0	0	1	0.0:1	0.001	0.02
Total	10,952	15,537	26,567	4485	15,434	2524	75,499	2.4:1	100	–
Taxa	9	9	10	9	9	9	11	–	–	–
%	14.506	20.579	35.189	5.940	20.443	3.343	100	–	–	–
H′	0.18	0.25	0.27	0.38	0.36	0.42	–	–	–	–

H′, Shannon diversity index, SISA, standardized index of species abundance

^a^Environments. P1A, P1B, P1C: collection sites in forested area at heights of 12 m (P1A), 6 m (P1B) and 1 (P1C). P2, P3, P4: collection sites at forest boundary (P2), rural peridomestic environment (P3) and peridomestic environment of the residential condominium complex (P4)

*Psychodopygus wellcomei* was more abundant in the forest (P1C) and boundary areas (P2) (Fig. 2), in addition to occurring in anthropized environments, such as the rural peridomestic (P3) and condominium (P4), albeit not as abundantly as in the forest and boundary areas (Fig. 2).

The highest abundance of *Lu. longipalpis* occurred in the the rural peridomestic environment (P3) (SISA = 0.831), where it was the second most abundant species. It also occurred in other ecotopes, but at a lower abundance (Fig. 2).

Analysis of the most abundant species in forest and anthropized environments (Fig. 2) revealed that *Ev*. *walkeri* and *Ps. wellcomei* occurred predominantly in the former and *Lu. longipalpis* primarily in the latter (Table 1).

### Vertical and horizontal stratification

The traps with the highest number of phlebotomines were those placed near the ground in forest areas, namely 26,567 collected specimens (35.2%) at P1C and 15,537 (20.6%) at P1B. In an anthropized area, the largest number of phlebotomines occurred in P3, a peridomestic rural area, with 15,434 specimens (20.4%) collected (Table 1).

*Evandromyia walkeri* was the eudominant species in all ecotypes studied (D% > 10%). *Psychodopygus wellcomei* was the eudominant species in traps located near the ground (P1B, P1C and P2), dominant species (5% < D > 10%) in tree canopies, subdominant species (2% < D > 5%) and occasional (1% < D > 2%) in traps at P3 and P4, respectively (i.e. in anthropized areas) (Table 2).

**Table 2:** Dominance index of phlebotomine species collected (2013–016) in different ecotopes, three vertical strata (P1A, P1B, P1C) and horizontal strata (P2, P3, P4), in Nísia Floresta, Rio Grande do Norte state, Brazil

Species	Ecotopes					
	Forest			Anthropized areas		
	P1A (%)	P1B (%)	P1C (%)	P2 (%)	P3 (%)	P4 (%)
*Evandromyia walkeri*	88.72^a^	82.00^a^	79.19^a^	72.69^a^	75.97^a^	67.55^a^
*Psychodopygus wellcomei*	9.63^b^	15.52^a^	18.32^a^	18.10^a^	2.85^c^	1.47^d^
*Evandromyia evandroi*	0.92^e^	1.22^d^	1.36^d^	5.62^b^	5.07^b^	21.43^a^
*Lutzomyia longipalpis*	0.05^e^	0.12^e^	0.23^e^	1.27^d^	13.62^a^	4.20^c^
*Evandromyia lenti*	0.08^e^	0.12^e^	0.15^e^	1.14^d^	2.12^c^	4.87^c^
*Psathyromyia brasiliensis*	0.33^e^	0.76^e^	0.52^e^	0.20^e^	0.01^e^	0.12^e^
*Psathyromyia abonnenci*	0.17^e^	0.15^e^	0.08^e^	0.09^e^	0.21^e^	0.08^e^
*Sciopemyia sordellii*	0.07^e^	0.06^e^	0.07^e^	0.54^e^	0.08^e^	0.12^e^
*Micropygomyia schreiberi*	0.02^e^	0.05^e^	0.06^e^	0.33^e^	0.06^e^	0.16^e^
*Nyssomyia whitmani*	0.00^e^	0.00^e^	0.01^e^	0.00^e^	0.00^e^	0.00^e^
*Evandromyia sallesi*	0.00^e^	0.00^e^	0.00^e^	0.02^e^	0.00^e^	0.00^e^
Total	100	100	100	100	100	100

^a^Eudominant species

^b^Dominant species

^c^Subdominant species

^d^Occasional species

^e^Rare species

*Luzomyia longipalpis* phlebotomines were rare (D < 1%) in forest environments (P1A, P1B and P1C), occasional at the edge (P2), eudominant in the peridomestic rural area and subdominant in the residential community (P4) (Table 2).

The occurrence of *Ev. vandroi* was rare in the canopy (P1A) and only occasionally near the ground in a forest area (P1B and P1C). Dominance rose as the distance from this area increased, with this species becoming dominant at the forest edge (P2) and in the peridomestic rural area (P3) and eudominant, along with *E. walkeri*, in the residential community. The other species were mostly rare, except for *Ev. lenti*, whose presence was occasional in P2 and subdominant in P3 and P4 (Table 2).

Despite the significant abundance of phlebotomines in all the forest traps, these insects were most active near the ground (P1C) and in the tree canopy. The differences were significant between the traps near the ground and in the canopy (P1A) (Mann-Whitney U-test:* U* = 23,* Z* = 2.46,* P* = 0.0069), and between the midpoint (P1B) and canopy (P1A) (Mann-Whitney U-test:* U* = 35.5,* Z* = 1.64,* P* = 0.0503). No significant difference was found between P1B and P1C (*P* = 0.1252).

### Seasonal and daily phlebotomine activity

The highest abundance of* Ev. walkeri* was observed in January and February, which is the transition period between the dry and rainy seasons.

Pearson’s correlation test indicated that total phlebotomine abundance was positively correlated only with temperature (*r* = 0.39,* P* = 0.019); rainfall (*r* = − 0.15,* P* = 0.38) and relative humidity (*r* = − 0.1158,* P* = 0.50) were not correlated with insect abundance. The estimate using simple linear regression analysis revealed that for each degree increase in temperature, an average of 888.5 more phlebotomine specimens occurred [Coef. (b) = 888.5,* P* = 0.019].

In separate analyses of the five main species collected, *Ev. walkeri* abundance was correlated only with temperature (*r* = 0.43,* P* = 0.009), such that for each degree increase, there was an average increase of 904.1 phlebotomines [Coef. (b) = 904.08, * P* = 0.009]. The species *Ev. evandroi* and *Lu. longipalpis* showed no significant correlation between abundance and the meteorological variables (*P* > 0.05) assessed. *Evandromyia lenti* abundance was correlated only with temperature (*r* = 0.3751, * P* = 0.02); that is, for each degree increase, there was an average of 10.3 more individuals of this species [Coef. (b) = 10.3, * P* = 0.02]. *Psychodopygus wellcomei* exhibited a significant correlation between abundance and the variables rainfall (*r* = 0.47, * P* = 0.004) and relative humidity (*r* = 0.53,* P* = 0.0009); the relation with rainfall was such that for each additional millimeter of rain, there was an average increase of 1.2 individuals of the species [Coef. (b) = 1.23, * P* = 0.004], while for relative humidity, abundance increased by an average of 73 individuals for each percentage rise in humidity [Coef. (b) = 73.05, * P* = 0.0009].

Although none of the meteorological variables were correlated with phlebotomine abundance at the moment of collection, it was considered possible that cross-correlation analysis might reveal the influence and dynamics of the time interval that were not observed in the previous analysis. Thus, the total number of phlebotomines from the five most abundant species was analyzed in relation to the rainfall, temperature and relative humidity of previous months. Cross-correlation analysis revealed positive correlations between phlebotomines and rainfall in the insect collection month (Lag 0), and between 1 (Lag-1) and 2 (Lag-2) months before collection. The correlation observed with temperature was negative and significant between 3 and 5 months before collection (Lag-3, Lag-4 and Lag-5). The relative humidity 4 and 5 months before insect collection month (Lag-4, Lag-5) was directly correlated with the number of phlebotomines.

Analysis of the lagged correlation between rainfall and the number of phlebotomines collected revealed significant results between the species *Ev. evandroi* (Lag-4 and Lag-5), *Ev. lenti*, *Ev. walkeri* and *Lu. longipalpis* and rainfall during 4 and 5 months prior to collection (Lag-4 and Lag-5), while *Ps. wellcomei* demonstrated a significant positive correlation with rainfall during the collection month (Lag 0) and the previous month (Lag-1) (Table 3).

**Table 3:** Time lag analysis of phlebotomine abundance and rainfall of the five most abundant species captured in CDC traps, according to the standardized index of species abundance

Species	Time lag period^a^											
	0	*P*	−1	*P*	−2	*P*	−3	*P*	−4	*P*	−5	*P*
*Evandromyia walkeri*	− 0.246	0.148	0.065	0.712	0.336	0.052	0.212	0.236	0.432	0.013	0.535	0.002
*Psychodopygus wellcomei*	0.471	0.004	0.345	0.042	0.006	0.972	− 0.192	0.286	− 0.103	0.573	− 0.292	0.111
*Evandromyia evandroi*	− 0.035	0.840	0.072	0.679	0.273	0.118	0.264	0.137	0.579	0.001	0.460	0.009
*Lutzomyia. longipalpis*	− 0.030	0.861	− 0.011	0.952	0.097	0.584	0.007	0.967	0.368	0.038	0.585	0.001
*Evandromyia lenti*	− 0.031	0.859	0.128	0.463	0.294	0.091	0.321	0.069	0.551	0.001	0.478	0.007

^a^Lag 0: insect collection month; Lag-1, -2, -3, -4, -5: 1, 2, 3, 4 and 5 months preceding collection, respectively

A significant time lag was also observed when comparing the occurrence of some phlebotomine species with the temperatures of previous months. *Evandromyia evandroi* showed a significant correlation with the temperatures recorded between 1 and 5 months before collection (Lag-1 to Lag-5). For *Ev. lenti*, the correlation was significant with the temperature at collection time (Lag 0), and between 1 and 5 months before collection (Lag-1 to Lag-5); for *Ev. walkeri*, the correlation was significant with temperature at collection time (Lag 0), and at 1, 2 and 5 months before collection (Lag-1, Lag-2 and Lag-5); *Lu. longipalpis* showed a negative correlation with temperature at 5 months before collection time (Lag-5) and *Ps. wellcomei* with temperature between 1 and 2 months before collection time(Lag-1 and Lag-2) (Table 4).

**Table 4:** Time lag analysis of the relation between phlebotomine abundance and temperature of the five most abundant species captured in CDC traps

Species	Time lag period											
	0	*P*	−1	*P*	−2	*P*	−3	*P*	−4	*P*	−5	*P*
*Evandromyia walkeri*	0.424	0.010	0.4808	0.003	0.4618	0.006	0.312	0.077	− 0.113	0.540	− 0.597	< 0.001
*Psychodopygus wellcomei*	− 0.1223	0.477	− 0.444	0.008	− 0.4363	0.010	− 0.353	0.044	− 0.246	0.174	− 0.162	0.384
*Evandromyia evandroi*	0.2914	0.085	0.3824	0.023	0.3203	0.065	0.127	0.482	− 0.287	0.111	− 0.621	< 0.001
*Lutzomyia longipalpis*	0.025	0.883	0.082	0.641	0.158	0.374	0.091	0.616	− 0.173	0.344	− 0.416	0.020
*Evandromyia lenti*	0.3695	0.027	0.389	0.021	0.32	0.065	0.126	0.484	− 0.313	0.081	− 0.675	< 0.001

The species *Ev. evandroi*, *Ev. walkeri* and *Lu. longipalpis* showed a significant correlation with the 4 and 5-month lag time (Lag-4 and Lag-5). For *Ev. lenti*, these relations were observed between 3 and 5 months before collection time (Lag-3, Lag-4 and Lag-5), while for *Ps. wellcomei*, the lagged relations for relative humidity were observed at collection time, and at 1, 4 and 5 months before collection (Lag-1, Lag-4 and Lag-5) (Table 5).

**Table 5:** Time lag analysis of the relation between phlebotomine abundance and relative umidity of the five most abundant species captured in CDC traps

Species	Time lag period											
	0	*P*	−1	*P*	−2	*P*	−3	*P*	−4	*P*	−5	*P*
*Evandromyia walkeri*	− 0.213	0.211	0.053	0.763	0.255	0.146	0.293	0.098	0.483	0.005	0.520	0.003
*Psychodopygus wellcomei*	0.527	0.001	0.346	0.042	0.046	0.797	− 0.201	0.263	− 0.366	0.039	− 0.422	0.018
*Evandromyia evandroi*	− 0.213	0.211	0.053	0.763	0.255	0.146	0.293	0.098	0.483	0.005	0.520	0.003
*Lutzomyia longipalpis*	− 0.123	0.474	− 0.059	0.738	0.044	0.803	0.194	0.279	0.401	0.023	0.406	0.024
*Evandromyia lenti*	− 0.067	0.696	0.104	0.554	0.294	0.092	0.395	0.023	0.586	<0.001	0.463	0.009

**Fig. 3:**
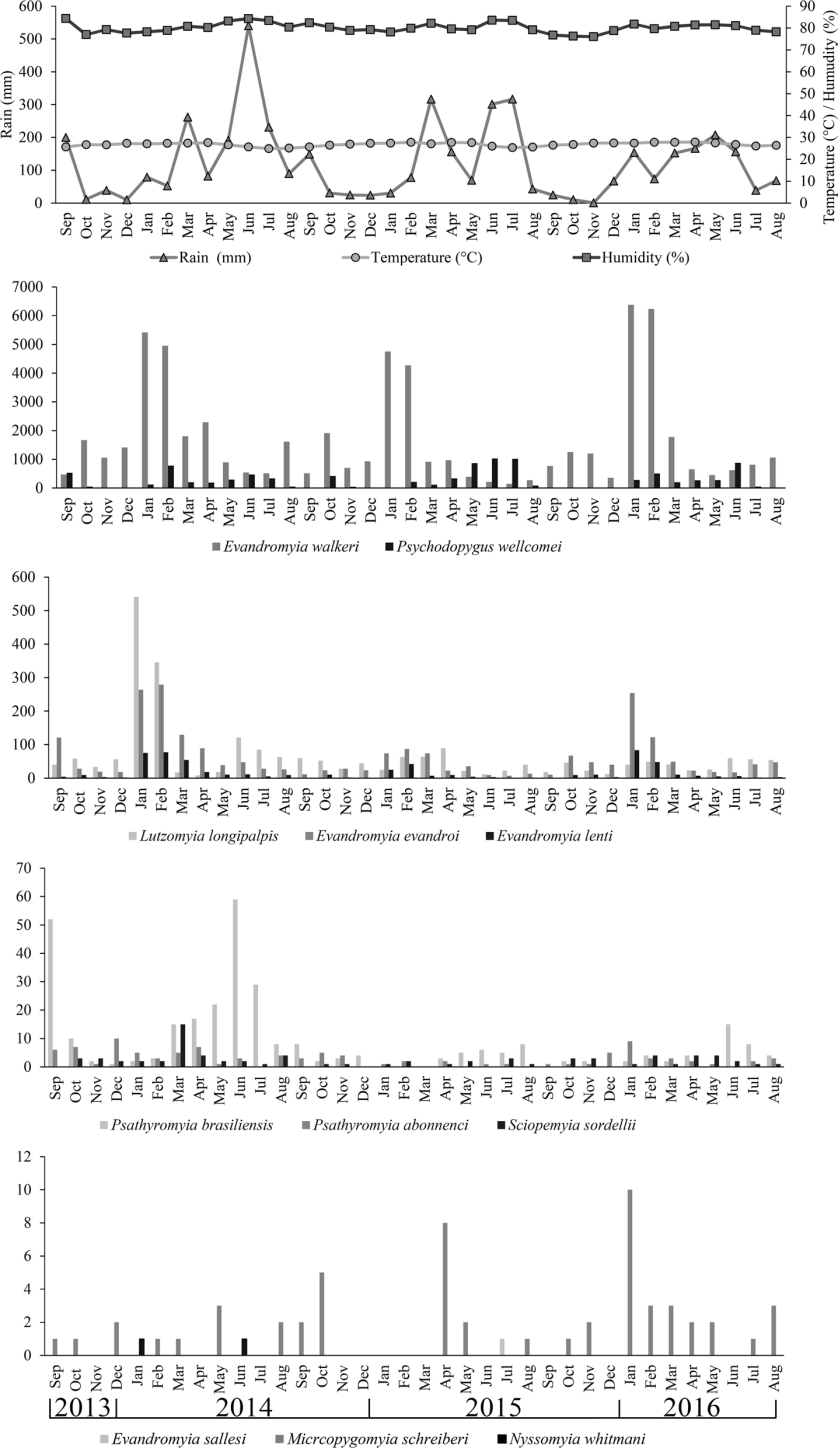
Monthly occurrence of the species and climate variables in the Atlantic Forest conservation unit of Nisia Floresta, Rio Grande do Norte, Brazil

Seasonal species occurrence varied during the 3 collection years. Peak abundance of *Ev. walkeri*,* Ev. lenti* and *Ev. evandroi* was always observed in January and February. The highest occurrence of *Ps. wellcomei* was observed in the rainy season (May, June and July), revealing marked seasonality during the three years, but this species was essentially absent in the dry season (Fig. 3).

Collections using Shannon traps during three periods of 24 consecutive hours resulted in the capture of 3914 phlebotomines belonging to the species *Ps. wellcomei*, *Ev. walkeri*, *Ev. evandroi* and *Lu. longipalpis* were collected in the 72-h sampling effort. The most abundant of these was *Ps. wellcomei*, with 3598 individuals (91.93%) (SISA = 0.92), followed by *Ev. walkeri*, with 306 individuals (7.82%) (SISA = 0.87). Only eight (0.20%; SISA = 0.47) and two (0.05%; (SISA = 0.44) individuals of *Ev. evandroi* and *Lu. longipalpis*, respectively, were captured (Table 6).

**Table 6:** Shannon trap captures during three collections, one per month, each for 24 consecutive hours

Shannon trap captures	Collection month			Total number	Males (*n*)	Females (*n*)	Male:female ratio	Percentage distribution	SISA
	June 2016	August 2016	October 2016						
Species									
* Psychodopygus wellcomei*	3470	127	1	3598	283	3315	0.08:1	91.93	0.92
* Evandromyia walkeri*	32	57	217	306	205	101	2.03:1	7.82	0.87
* Evandromyia evandroi*	0	7	1	8	8	0	−	0.20	0.47
* Lutzomyia longipalpis*	0	1	1	2	0	2	0.0:1	0.05	0.44
Total	3502	192	220	3914	496	3418	0.14:1	100	−
%	89.47	4.91	5.62	100	12.67	87.33	–	–	–
H′	0.125	0.339	0.037	–	–	–	–	–	–

**Fig. 4:**
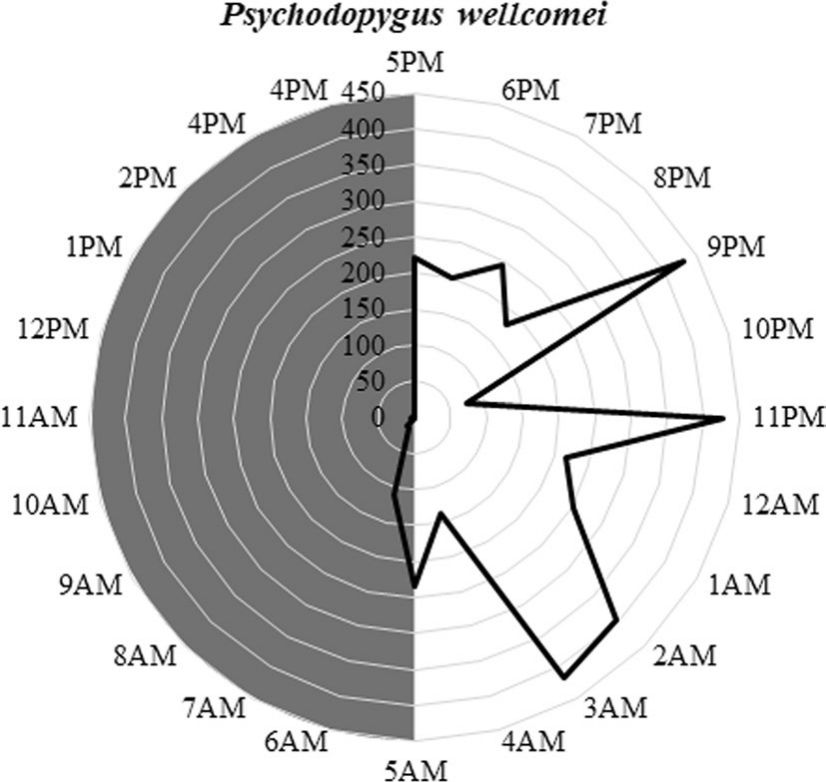
Hourly occurrence of *Psychodopygus wellcomei,* the most abundant species in three 24-hour collections in the Atlantic Forest Conservation Unit of Nisia Floresta, Rio Grande do Norte, Brazil

The numerical daily *Ps. wellcomei* occurrence was concentrated in the two twilight periods, as well as early and late at night (Fig. 4). The largest number of specimens were captured in June (89.47% of the total). Similar numbers were recorded in August, the month with the greatest diversity (Table 6), and in October, but the number was much less than during the first capture. The hours of greatest activity varied between the sampling months, with significant differences (Kruskal-Wallis H-test:* H* = 39.5829,* GL* = 23,* P* = 0.0171).

## Discussion

Of the 75,499 sand flies belonging to 11 species caught with CDC traps, the most abundant were *Ev. walkeri*, *Ps. wellcomei* and *Lu. longipalpis*. The last two are vectors of the protozoa *Leishmania infantum* *and L braziliensis*, etiological agents of CL and VL in Brazil, respectively. A total of 3914 phlebotomines of the species *Ps. wellcomei*, *Ev. walkeri*, *Ev. evandroi* and *Lu. longipalpis* were captured in the Shannon traps. Analysis of the ecological relationships and behavior of the species revealed that in both collecting systems *Ps. wellcomei* stands out as dominant species in forest and degraded environments while *Lu. longipalpis* is present only rarely in the forest environment and is dominant in degraded environments. This result is reflected in the potential risk of transmission in an endemic area, although *Ev. walkeri* was the most abundant species in the area during the 3 years of the study.

The most abundant species in the CDC trap in all the ecotopes under study was *Ev. walkeri*, accounting for 79.7% of captures, while *Lu. longipalpis* accounted for 3.1% of captures. Comparison with an earlier study conducted in an ecologically isolated area in the same municipality, where *Ev. walkeri* represented only 7.46% of the phlebotomines captured and *Lu. longipalpis* 64.47% [15], shows a change in species composition, making fine-scale bioecological investigations important. Although *Ev. walkeri* is not associated with *Leishmania* transmission in the region, the species was recently found to be naturally infected with *L. braziliensis* in Acre State, northern Brazil [30]. Of the species collected in our study, *Lu. longipalpis* is the main vector of *L. infantum*, causing VL throughout the country, particularly in the northeastern region, with a large number of cases, resulting in death in up to 10% of the total cases [1, 31]. In the present study, *Lu. longipalpis* was poorly represented, when compared to an earlier study in which it accounted for > 70% of the phlebotomines collected, also in the municipality of Nísia Floresta, but in a peridomestic environment [32]. Changes in the composition and population dynamics of phlebotomines in the study area suggest phlebotomine species succession. All of the species exhibited preferential ground-level behavior, with *Lu. longipalpis* continuing to be found primarily in degraded or anthropized areas.

*Psychodopygus wellcomei* was dominant in the June collection, when 92% of all the individuals of this species were captured, a finding related to the rainy season, characterized by relatively higher relative humidity and lower temperatures. Given that the physiological, biochemical and behavioral processes of living beings are influenced by daily and seasonal variations, it is important to underscore the greater occurrence of *Ps. wellcomei* in the rainy season in Nísia Floresta, which is likely entering into diapause in the dry season, as reported in other studies conducted in the Brazilian Amazon and northeastern Brazil [33–35]. With respect to vertical stratification, *Ps. wellcomei* was observed in the tree canopies, which may be associated with the acquisition of carbohydrates in plant species or the female’s search for a blood meal in arboreal animals, such as birds, *Callithrix jacchus* (common marmosets) and *D. albiventris* (opossums), the species found in the area. Different degrees of forest cover may also reflect phlebotomine behavior. A large leaf index area provides suitable conditions for phlebotomine activities, even influencing natural flagellate infection rates [36]. Despite its greater abundance in the preserved forest area (Fig. 2), the presence of this species approximately 20 m from the edge of the conservation unit suggests a selection of new habitats and consequent expansion of the vital area of this species.

The correlation between the climate parameters and the number of phlebotomines reveals the influence of the former over longer time periods on phlebotomine abundance. The lagged correlation between rainfall and the phlebotomines collected showed significant results between the species *Ev. evandroi*, *Ev. lenti*, *Ev. walkeri* and *Lu. longipalpis* and rainfall during the 4 and 5 previous months, while *Ps. wellcomei* demonstrated a significant positive correlation with the rainfall of the collection month and the month directly preceding the collection time. Rainfall in the region is irregular, and with a low index, the development peaks of immature individuals likely occur in microhabitats when soil humidity and temperature conditions are favorable, as we observed in the laboratory. *Psychodopygus wellcomei* shows a close relationship with rainfall, revealing a strong presence in the rainy months over a 3-year study period.

In Rio Grande do Norte, despite the relatively low incidence of CL cases when compared to other Brazilian states, the observations and results obtained are important because *Ps. wellcomei* has become more abundant in recent years in forest fragments, predominating in forest environments [37] close to or within areas in which people and animals are active during the day. Studies in Amazonia have also shown that infected females are more frequently captured during the day than at night, making transmission by this phlebotomine species more common during the day [34, 38–41]. *Nyssomyia whitmani*, which is infrequently observed in the area, was found in the forest and anthropized area. This species requires our attention due to the species of *Leishmania* found in this phlebotomine and its involvement in CL and VL. With respect to *Ev. lenti*, there is a record of natural infection in southeastern and northeastern Brazil [42–44].

Although it was not possible to perform an analysis of infection, the natural investigation of infection by *Leishmania* associated with cases of CL is important in all the areas of occurrence of the species. *Psychodopygus wellcomei* is highly anthrophilic, but its epidemiological importance is restricted to CL cases in the Amazon region; it association with cases of CL has yet to be confirmed in the northeastern region [34, 37–40]. The Shannon trap 24-h captures reveal a large abundance of *Ps. wellcomei*, primarily females, which likely approach the traps not only attracted by the light source but also due to high species anthropophilia, being attracted by exhaled odors and CO_2_, as noted by their attempts to feed on the researchers [37, 45, 46]. Among the species collected, the vectorial capacity or competence of *Lu. longipalpis* for *L. infantum* and of *Ps. wellcomei* and *Ny. whitmani* for *L. braziliensis* has been described earlier [34], as has the presence of circulating parasites or detected DNA of some *Leishmania* species in *Ev. walkeri*, *Ev. evandroi*, *Ev. lenti*, *Sc. sordellii* and *Ev. sallesi* [2, 30, 34, 42–53] (Table 7). Despite the small sample size used to draw firm conclusions regarding daily phlebotomine activity patterns, it is important to underscore that the daily activity pattern of *Ps. wellcomei* is similar to that found in another biome, Amazonia, where the species feeds avidly and remains active during the day, primarily in cloudy weather [54]. In our study, *Ps. wellcomei* females were more active between 5 p.m. and 5 a.m., likely in search of hosts, with a lower occurrence outside of these hours. This information may be useful in preventing CL, since armed with this knowledge, people can avoid the occurrence sites of the vector at peak times or use preventive measures, such as repellent or adequate clothing.

**Table 7:** Vetorial capacity/competence and circulating parasite/detected DNA in the captured phlebotomines species

Species	Vectorial capacity/competence	Circulating parasite/detected DNA
*Evandromyia walkeri*	Unknown^a^	*L*. *braziliensis* [30]
*Psychodopygus wellcomei*	Yes, for *L*. *braziliensis* [34]	*L*. *braziliensis* and *L*. *infantum* [34, 51]
*Evandromyia evandroi*	Unknown	*L*. *infantum* and *L*. *lainsoni* [42, 51]
*Lutzomyia longipalpis*	Yes, for *L*. *infantum* [34]	*L*. *infantum*, *L*. *shawi*, *L*. *mexicana, L*. *lainsoni*, *L*. *amazonensis*, *L*. *guyanensis* and *L*. *braziliensis* [34, 42, 44, 48, 51–53]
*Evandromyia lenti*	Unknown	*L*. *infantum* and *L*. *braziliensis* [43, 44, 49]
*Psathyromyia brasiliensis*	No evidence^b^	None detected
*Psathyromyia abonnenci*	No evidence	None detected
*Sciopemyia sordellii*	Unknown	*L*. (*Leishmania*) sp. and *L*. *infantum* [47, 50, 51]
*Micropygomyia schreiberi*	No evidence	None detected
*Nyssomyia whitmani*	Yes, for *L*. *braziliensis* [34]	*L*. *braziliensis*, *L*. *lainsoni*, *L*. *amazonensis*, *L*. *infantum* and *L*. *shawi* [2, 34, 43, 44, 49, 51–53]
*Evandromyia sallesi*	Unknown	*L. braziliensis* complex [50]

^a^Unknown refers to species that had parasites or DNA detected, but for which the relevance as well as the vectorial capacity and competence of the species in the natural transmission of the parasites is not known

^b^No evidence refers to species that had no DNA or parasites detected

*Evandromyia evandroi* was more abundant in peridomestic environments and in conserved forest, while *Ev. lenti* was more abundant at the edge of the forest and in peridomestic environments (Fig. 2), primarily on the rural property, which shows that it is adapted to modified environments. These species are less abundant in the peridomestic area of the residential condominium, likely because it was recently constructed and surrounded by walls that can act as flight barriers, while in the rural setting, the area is open and shaded, with possible food sources, such as fruit trees, chickens, ducks, dogs and horses.

Our observations on phlebotomines in the region reveal that those which occur during the day generally land on the hosts at twilight, remaining throughout the night and disappearing at daybreak, with the exception of female *Ps. wellcomei*, which remain active during the day, albeit at lower densities than at night, and absent only between 1 and 2 p.m. We found the phlebotomine *Ev. walkeri* to be active only at twilight and at night, with peak occurrences between 11 p.m. and 3 a.m.. The varying activity times of these insects may be related to competitive strategies for food sources. This competition occurs when different species colonize the same space and may become more intense as food sources are depleted [55, 56].

Finally, in relation to potential vectors, our analyses reveal greater abundance of *Ps. wellcomei*, a species with vectorial capacity/competence for *L. braziliensis*, in the conserved forest (Fig. 2), although it also occurs in the peridomestic environment where *L. longipalpis*, a vector species of *L. infantum*, is more abundant (Fig. 2). It is notable that it occurs in association with people and domestic animals, which calls attention to the risk of transmission. The expansion of human activities, with environmental impacts and changes in land use, produces new potentially occupiable niches, affects fauna composition and the vector reservoir and parasite behavior, in addition to their interrelationships, which may lead to changes in local leishmaniasis epidemiology.

## Conclusions

This species abundance study reveals the predominance of *Ev. walkeri* in the forest and anthropized areas, followed by *Ps. wellcomei*, which exhibited the same distribution pattern, predominantly in the rainy season. The structure of the phlebotomine community is influenced by abiotic factors, interactions with plant and animal species and the degree of environmental disturbance. Conditions for *L. braziliensis* and *L. infantum* transmission to occur exist in the study area, namely primitive parasite reservoirs, susceptible vertebrate hosts, vectors and environmental conditions favorable to their development, in addition to being a region endemic for VL and CL. The seasonality and daily activity observed for *Ps. wellcomei* and *Lu. longipalpis* modulate the relation between the vector and vertebrate host and consequently the risk of infection. As such, these areas need to be protected and surrounding areas containing houses or agricultural activities in urban, periurban or rural settings should be permanently monitored to prevent the onset of VL or CL transmission cycles and outbreaks.

## Data Availability

Data supporting the study are included within the article.

## Abbreviations

CL: Cutaneous leishmaniasis
CU: Conservation Unit
INMET: National Institute of Meteorology
SISA: Standardized index of species abundance
VL: Visceral leishmaniasis
